# Risk of Recurrence and Cancer Stem Cell Marker CD133 Expression Vary in Males Versus Females with Papillary Thyroid Cancer

**DOI:** 10.1245/s10434-025-17256-2

**Published:** 2025-04-11

**Authors:** Jeremy Chang, Eyas M. Alzayadneh, Anand Rajan, Andy Tran, Ronald J. Weigel, Anna C. Beck

**Affiliations:** 1 Department of Surgery, University of Iowa Hospitals and Clinics, Iowa City, IA USA; 2 Department of Pathology, University of Virginia, Charlottesville, VA USA; 3 Department of Pathology, University of Iowa Hospitals and Clinics, Iowa City, IA USA; 4 Department of Surgery, University of Wisconsin - Madison, Madison, WI USA

## Abstract

**Background:**

Molecular profiling has refined the identification of pathologic subtypes in other cancers, but is not currently used for prognostication in papillary thyroid cancer (PTC). The cancer stem cell marker, CD133, is a glycoprotein associated with tumor initiation and radioresistance in PTC, but its role in prognostication continues to be defined. This study sought to define the association between CD133 expression in PTC and recurrence-free survival (RFS).

**Methods:**

All the patients at a single institution with PTC who underwent thyroidectomy from 2008 to 2011 were identified through an electronic medical record query. Immunohistochemistry was performed for CD133, and the H-score was calculated. Receive operating characteristic (ROC) curves were used to identify the optimal cutoff for CD133 expression.

**Results:**

Overall, 110 consecutive patients were identified, and 12% had a biopsy-proven recurrence during a median follow-up period of 10.1 years (interquartile range [IQR], 8.3–11.5 years). The median H-score did not differ significantly between the patients who experienced recurrence (74; IQR, 59–81) and those who did not (70; IQR, 51–89). An H-score of 55 or higher identified patients who had a recurrence with 92% sensitivity. Among the patients with a CD133 H-score of 55 or higher, males had a significantly worse RFS than females (*p* = 0.005), but RFS did not differ between males and females with a CD133 H-score lower than 55 (*p* = 0.739).

**Conclusions:**

A clear role of CD133 in prognostication has not been defined to date. Expression of CD133 and its association with survival varies between males and females, with stratification of recurrence risk more prominent in males.

**Supplementary Information:**

The online version contains supplementary material available at 10.1245/s10434-025-17256-2.

Papillary thyroid carcinoma (PTC) is a well-differentiated thyroid cancer with excellent overall survival. However, locoregional recurrences still occur, making risk stratification by risk of recurrence important to guide management recommendations.^1–3^ The American Thyroid Association (ATA) guidelines categorize well-differentiated thyroid cancer as high, intermediate, and low risk for recurrence based on a multitude of clinical features as well as the mutation status of *BRAF* and *TERT.*^4^ Molecular profiling has refined the identification and prognostication of pathologic subtypes in other cancers, but is limited in papillary thyroid cancer. Rather, in the setting of thyroid cancer, molecular testing has primarily focused on risk stratification of thyroid nodules,^5–7^ and testing for somatic mutations in *BRAF* and *TERT* is not performed for all patients with a new diagnosis of PTC.^4^ The current accepted prognostication for PTC has focused on clinical features, including the ATA guidelines for risk stratification, which have been well-validated.^4,8,9^

Cancer stem cells have been increasingly better understood in their role for tumor initiation and progression.^10–13^ As a transmembrane glycoprotein, CD133 is well recognized as a cancer stem cell marker in many cancer types, including thyroid cancer.^14,15^ In PTC, it has been demonstrated that CD133-expressing cells are responsible for tumor initiation and may be radioresistant, allowing them to survive radioiodine therapy.^16–18^ Whereas other cancer stem cell markers have shown associations between level of expression and prognosis in PTC, the ability to use CD133 as a prognostic marker in thyroid cancer is unclear.^19–23^

Previously, we characterized higher expression of CD133 by immunohistochemistry (IHC) in the more aggressive tall cell variant of PTC and demonstrated that a gene signature, which included the *PROM1* gene encoding CD133 characterizing tall-cell-variant PTC, was associated with worse disease-free survival (DFS) when applied to classic PTC.^24^ However, assessment of tumors using IHC is a more readily available technique for clinicians than gene expression profiling. Thus, our aim in the current study was to assess the application of IHC assessment of CD133 expression in patients with classic PTC as a method to prognosticate outcomes beyond the clinical variables and *BRAF/TERT* mutational analysis used by the ATA guidelines. We hypothesized that using the level of expression of the cancer stem cell marker, CD133, by IHC in classic PTC may improve risk stratification compared with clinical features alone.

## Methods

After institutional IRB approval, consecutive patients at the University of Iowa Hospitals and Clinics with PTC who underwent total thyroidectomy from 2008 to 2011 were identified through an electronic medical record query. The timing of the study cohort was decided to allow for robust follow-up evaluation, with the potential of all patients to have at least a 10-year follow-up period given the indolent nature of PTC.

We defined patients as having a recurrence if local or distant disease was identified with pathologic confirmation at least 1 year after total thyroidectomy.^25^ Any recurrence less than 1 year after total thyroidectomy was defined as persistent disease, and these patients were excluded. Thyroglobulin levels were not used to define recurrent or persistent disease, but were used to calculate ATA risk. Patients were excluded if they were younger than 18 years, had fewer than 5 years of documented follow-up evaluation, had metastatic disease at the time of presentation, or had persistent disease identified pathologically within 1 year after total thyroidectomy. Only those with classic or follicular-variant PTC were included in the study. More aggressive subtypes, including tall-cell PTC, were excluded. Any patient without available primary tumor blocks was excluded. The Cancer Genome Atlas (TCGA) Thyroid Cancer database, which is publicly available, was accessed through xenabrowser.net.

All tumor blocks included were reviewed by authors Eyas M. Alzayadneh and Anand Rajan to confirm diagnosis. Only primary tumors were used for analysis. The primary tumors were stained by IHC for CD133 (Abcam, Cambridge, MA, USA). After IHC, the H-score was calculated using 3D Histech Quantcenter, a digital analysis platform with intensity measurement capacity using the DensitoQuant algorithm.^24,26^ At least three areas of tumor were selected manually to appropriately represent the staining distribution of each tumor, and H-scores were calculated according to the standard method as (1 × proportion of pixels with weak staining) + (2 × proportion of pixels with moderate staining) + (3 × proportion of pixels with strong staining), with a range of 0 to 300 and with higher scores indicating greater protein expression. Sites of analysis were identified by Eyas M. Alzayadneh, then independently reviewed by Anand Rajan to confirm that they were representative. Both authors were both blinded to the clinical data, including the recurrence status, of all the patients. The final H-scores were calculated byAnand Rajan.

Statistical analysis was performed with GraphPad Prism and IBM SPSS Statistics version 29.0.0.0. Pearson chi-square and independent samples *t* tests were used as appropriate. Receiver operating characteristic (ROC) curves were used to identify the optimal cutoff for CD133 expression, with the goal of obtaining high sensitivity. The alpha value was defined as 0.05.

## Results

Overall, 110 patients with PTC who had a total thyroidectomy and met the inclusion criteria were identified: 91 (83%) females and 19 (17%) males. The median age was 46 years (interquartile range [IQR], 34–55 years), and 72.7% of the patients received radioactive iodine. Additional patient characteristics are detailed in Table 1.

**Table 1:** Patient characteristics of all patients and by recurrence status

	All patients (*n* = 110)		Nonrecurrent (*n* = 97)		Recurrent (*n* = 13)		*p* value
	N	%	N	%	N	%	
Median age: years (IQR)	46.0 (34–55)		46.0 (34–56)		52.0 (39–56)		0.944
Age category (years)							
< 55	79	71.8	69	71.1	10	76.9	0.663
> 55	31	28.2	28	28.9	3	23.1	
Sex							
Male	19	17.3	13	13.4	6	46.2	0.003
Female	91	82.7	84	86.6	7	53.8	
Race							
White	102	92.7	90	92.8	12	92.3	0.735
African American	0	0.0	0	0.0	0	0.0	
Asian	3	2.7	2	2.1	1	7.7	
Hispanic	3	2.7	3	3.1	0	0.0	
Other	1	0.9	1	1.0	0	0.0	
Histological subtype							
Classic	80	72.7	68	70.1	12	92.3	0.334
Follicular variant	18	16.4	18	18.6	0	0.0	
Classic + follicular variant	11	10.0	20	20.6	1	7.7	
Columnar cell variant	1	0.9	1	1.0	0	0.0	
ATA risk stratification							
Low	62	56.4	62	63.9	0	0.0	< 0.001
Intermediate	37	33.6	30	30.9	7	53.8	
High	11	10.0	5	5.2	6	46.2	
Any nodal disease	48	43.6	37	38.1	11	84.6	0.002
Extranodal extension	23	20.9	17	17.5	6	46.2	0.057
Lymphovascular invasion	18	16.4	14	14.4	4	30.8	0.312
Elevated thyroglobulin post-op	13	11.8	9	9.3	4	30.8	0.061
Stage							
I	99	90.0	89	91.8	10	76.9	0.094
II	11	10.0	8	8.2	3	23.1	
III	0	0.0	0	0.0	0	0.0	
Received RAI	80	72.7	67	69.1	13	100	0.019
Median CD133 H-score (IQR)	70.7 (52–87)		70.0 (51–89)		74.4 (59–81)		0.555
CD133 H score ≥ 55	80	72.7	68	70.1	12	92.3	0.091

IQR, interquartile range; ATA, American Thyroid Association; RAI, radioactive iodine

The median follow-up period was 10.1 years (IQR, 8.3–11.5 years), and 13 patients (11.8%) had a biopsy-proven recurrence. An additional 10 patients (9.1%) had elevated serum thyroglobulin levels postoperatively, but did not experience a biopsy-proven recurrence during a median follow-up period of 10.2 years (IQR, 9.8–10.8 years).

**Fig. 1:**
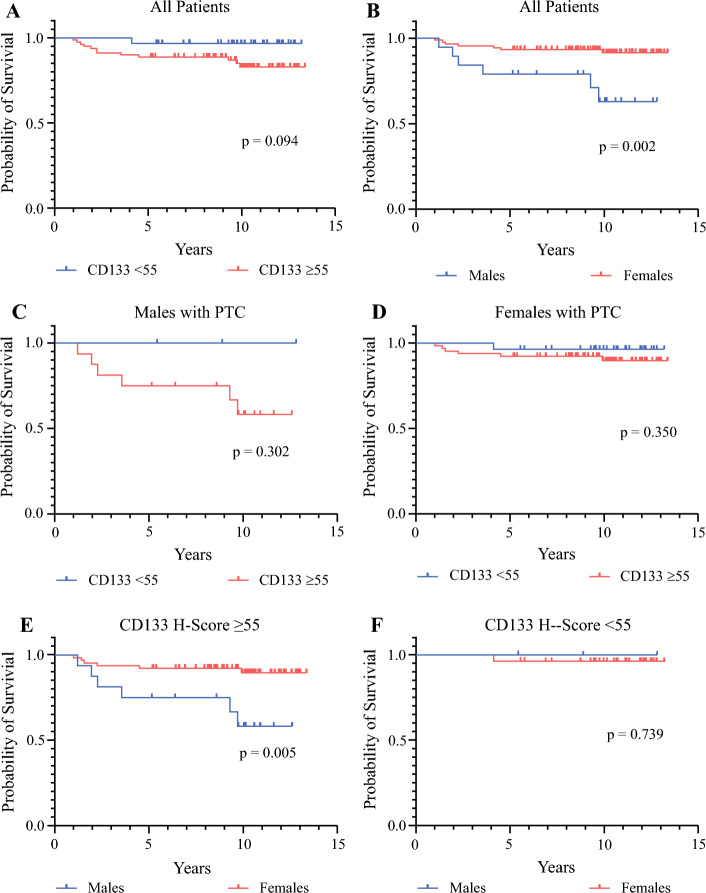
Recurrence-free survival (RFS) of patients with papillary thyroid cancer by level of CD133 expression. **A** High CD133 was not significantly associated statistically with worse RFS despite a trend toward worse RFS with an H-score of 55 or higher. **B** Males overall had worse RFS than females, **C**, **D** with CD133 expression stratifying males to a greater degree than females. **E** Males who had a CD133 H-score of 55 or higher had significantly worse RFS than females, **F** but males with a CD133 lower than 55 had an RFS similar to that of females

The median H-score in the IHC analysis of the overall cohort for CD133 was 70.7 (IQR, 52–87). As a continuous variable, CD133 was not significantly associated with recurrence-free survival (RFS), with a median H-score for CD133 of 74 (IQR, 59–81) among those who had recurrence and 70 (IQR, 51–89) among those who did not (*p* = 0.555). The area under the curve (AUC) with the ROC curve to assess the correlation between the CD133 H-score and recurrence was 0.530. To identify patients who had a recurrence, ROC curves were used to identify a cutoff in the CD133 H-score with a high sensitivity. A cutoff H-score of 55 was identified to have a sensitivity of 92% for all the patients. The CD133 H-score did not differ significantly between the males and females. The median H-score was 69.1 (IQR, 50–88) for the females versus 74.5 (IQR, 59–86) for the males (*p* = 0.604). No other clinical variables differed statistically between the patients with a CD133 of 55 or higher and those with a CD133 lower than 55 (Table 2), and no clinically significant trend was observed between CD133 H-score level and ATA risk classification (Table 3), demonstrating that level of CD133 expression may provide prognostic insight independent of any clinical variables. When RFS was compared, the patients who had a CD133 H-score of 55 or higher trended toward a worse RFS, although this difference did not reach statistical significance (Fig. 1A).

**Table 2:** Clinical variables by level of CD133 expression

	CD133 H-score				*p* value
	≥ 55		< 55		
	*n*	%	*n*	%	
Age (years)					
< 45	34	40.5	19	55.9	0.15
≥ 45	50	59.5	15	44.1	
Gender					
Male	17	20.2	3	8.8	0.18
Female	67	79.8	31	91.2	
Tumor size (cm)					
≤ 2	55	65.5	28	82.4	0.07
> 2	29	34.5	6	17.6	
Extracapsular extension					
No	62	73.8	28	82.4	0.32
Yes	22	26.2	6	17.6	
Multifocal					
No	41	48.8	22	64.7	0.12
Yes	43	51.2	12	35.3	
Lymphovascular invasion					
No	66	78.6	29	85.3	0.4
Yes	18	21.4	5	14.7	
Lymph node metastasis					
No	44	52.4	20	58.8	0.52
Yes	40	47.6	14	41.2

**Table 3:** Median H-score for CD133 by ATA risk classification

	ATA risk classification			*p* value
	Low (*n* = 62) *n* (range)	Intermediate (*n* = 37) *n* (range)	High (*n* = 11) *n* (range)	
All patients	65.2 (46–87)	76.0 (60–91)	65.1 (59–72)	0.047
Recurrence	–	76.0 (74–88)	65.1 (56–74)	0.103
No recurrence	63.2 (46–87)	77.5 (59–91)	65.1 (41–76)	0.197

We examined the association between RFS and the CD133 H-score in patient sub-cohorts to determine whether the association between CD133 and recurrence varied between the males and females (Fig. 1B–F). We observed that the males with a high CD133 trended toward a clinically, although not statistically, significant worse RFS, but this trend was not observed in the female patients (Fig. 1C and D). Interestingly, although RFS was significantly worse for the males than for the females overall (Fig. 1B; *p* = 0.002), when the males and females with a low CD133 H-score (< 55) were compared, the males with a low CD133 H-score had a similarly excellent RFS compared with the females (Fig. 1F). However, when only the patients with a high CD133 H-score of 55 or higher were examined, the males had a significantly worse RFS than the females (*p* = 0.005; Fig. 1E).

To further validate the findings in the current study, which demonstrated differences in association between CD133, sex, and recurrence, we used RNA expression levels and survival outcomes from the publicly available TCGA Thyroid Cancer database. The ROC curve for DFI was calculated using *PROM1* gene expression with an AUC of 0.57 (Fig. S1A). A cutoff of 5.9 log2 (norm_count+1) was identified with 74.6% specificity for DFI. The patients with a *PROM1* of 5.9 or higher were considered high-expressing, and those with a *PROM1* lower than 5.9 were considered low-expressing. In all the patients, DFI did not differ between high and low expressers (Fig. S1B). Although not statistically significant, the males with high PROM1 trended toward worse DFI (*p* = 0.08; Fig. S1C), but no trend was observed in the females (*p* = 0.97; Fig. S1D). Performance of PROM1 was examined by ROC curve for the males alone (Fig. S1E) and showed an improved AUC of 0.65 compared with an AUC of 0.55 for the females (Fig. S1F).

Additionally, we explored the association between *BRAF* mutation status, *PROM1* expression, and DFI. We identified that a correlation existed between the patients with a mutation in *BRAF* and a higher *PROM1* expression, and that in an analysis of patients separated by sex, this persisted only in the males (Fig. S2A–C). However, no clear trend in DFI was observed when the patients were stratified by both *BRAF* mutation status and *PROM1* expression (Fig. S2D–F).

## Discussion

Through IHC staining of well-differentiated papillary thyroid cancer for the cancer stem cell marker CD133, we identified that the association between CD133 expression and recurrence differed significantly between the male and female patients. Analysis of our entire cohort showed no statistically significant association between CD133 expression on IHC analysis of classic PTC tumors and RFS. However, although the males overall had worse RFS than the females, the male patients with low CD133 had an excellent RFS similar to that of the female patients with low CD133. These data suggest the existence of biologic factors that can be identified which stratify patients with PTC who appear at high risk clinically, and that these biologic factors likely vary between male and female patients.

A biologic difference between males and females in well-differentiated thyroid cancer have recently begun to be described. Clinically, when gender disparities in the setting of PTC have been examined, a recent meta-analysis found no difference in mortality between men and women.^27^ However, when specific biologic subtypes of well-differentiated thyroid cancer have been compared between males and females, recent studies have begun to identify differing prognostic indicators.

We identified that *PROM1* expression was higher in *BRAF-*mutated tumors in males within the TCGA Thyroid Cancer database, which may be partly why CD133 expression and prognosis differ in males. However, given that we found no clear correlation between *PROM1* expression, *BRAF* status, and DFI, this is currently unclear. Prior studies have identified that *BRAF* status and outcomes differ between males and females. Morand et al.^28^ compared mutational profiles between male and female patients with papillary and follicular thyroid cancer and found that although the absolute rate of point mutations was similar, mutations in *BRAF*^*V600E*^ and *TERT* occurred in males at a younger age than in females. Additionally, Wang et al.^29^ compared mortality in a large cohort of patients with papillary thyroid cancer and found that males with *BRAF*^*V600E*^ mutations had significantly higher mortality rates than females, but no difference in mortality rate was found between males and females in *BRAF* wild-type PTC.

Although the underlying cause of the difference in RFS between males with high expression of the cancer stem cell marker, CD133, and females could not be directly answered in the current associative study, preclinical data support the conclusion that the function cancer stem cells in thyroid cancer vary between patients with male biology and those with a female biology and may be related to estrogen exposure.^30,31^ To identify this, Zane et al.^31^ established xenografts in NOD/SCID mice using tissue from PTC tumors, then compared tumor growth between xenografts exposed to estrogen therapy and those not exposed. Tumor growth was significantly increased in those exposed to estrogen therapy. Additionally, the authors found that although cancer stem cell marker expression was enhanced in both males and females, the impact on cancer-associated signaling pathways varied. Tissue from males, when exposed to estrogen, had an upregulation of angiogenesis and epithelial-mesenchymal activation, both associated with aggressive behaviors in well-differentiated thyroid cancer, whereas tissue from females had an upregulation of all cell cycle phases, which the authors argued may make their tumors more inclined to be responsive to adjuvant treatments.^31^ These preclinical data offer possible insight into the clinical differences in RFS we observed in males and females with similar expression of the cancer stem cell marker, CD133, with higher expression of cancer stem cells in males possibly serving as a marker for the upregulation of more aggressive biologic pathways unique in patients with a male biology versus a female biology.

Clinically, the role of CD133 in papillary thyroid cancer continues to be defined. The level of CD133 expression in circulating tumor cells of patients with thyroid cancer has previously been shown to vary by level of differentiation of the tumor, with higher levels noted in poorly differentiated and anaplastic thyroid cancer.^20^ Others have found by IHC that CD133 is expressed in 61% of PTC tumors and not associated with any clinical features thought to be indicative of high recurrence risk, suggesting it may be a biologic marker that could be additive to clinical risk factors, similar to our findings in the current study.^21^

The importance of CD133 expression and treatment of thyroid cancer also continues to be established, with in vitro studies demonstrating radio-resistance in CD133+ papillary thyroid cancer cells and that CD133+ cells may be susceptible to novel therapeutic agents including all-trans retinoic acid, particularly in radio-resistant cells.^16,17^

Our findings support the conclusion that biomarkers can vary significantly between cancer subtypes and patient populations. Previously, CD133 expression by IHC was shown to be an unfavorable prognostic predictor in medullary thyroid carcinoma, with higher levels of expression associated with both worse progression-free survival and overall survival.^32,33^ Although the current study did not demonstrate a significant association in patients with PTC between level of CD133 expression by IHC and recurrence in our overall cohort, continued exploration of CD133 in sub-cohorts of patients including males, as our findings suggest, and patients with radioresistant tumors, based on the work of others as detailed earlier, may allow for the opportunity to better understand the role of CD133 and recurrence in PTC.

Potential next steps include analysis of CD133 expression in an expanded population of males with a diagnosis of PTC for potential prognostic significance. Alternatively, analysis of males and females with PTC on a multiomic level separately to identify a combination of biomarkers, in addition to CD133, also may allow for improved prognostication.

Limitations to our study include its retrospective nature, which limits the granularity of data collection. Due to the indolent nature of PTC, we had a small number of patients with recurrence, which limited the power of our analysis to detect statistic differences. Specifically, our relatively small number of patients with recurrence in the sub-cohort analysis likely limited the power of our analysis and therefore may have masked significant associations. Additionally, given the period from which we identified patients, although follow-up evaluation was optimized and likely improved our rate of detection of recurrence, the majority of the patients were treated with radioactive iodine, including all the patients who had a recurrence. This limited our ability to assess the association between CD133, receipt of RAI, and likelihood of recurrence.

## Conclusions

Risk for recurrence of papillary thyroid cancer may be identified through IHC using biologic markers, and may include the cancer stem cell marker CD133, although a clear role of CD133 in prognostication is not defined to date. Expression of CD133 and its association with survival vary between males and females, with stratification of recurrence risk more prominent in males. Additional work is necessary to determine the exact differences in expression of biomarkers such as CD133 between males and females with PTC to allow appropriate biomarkers to be implemented clinically.

## Supplementary Information

Below is the link to the electronic supplementary material.

Supplementary file1 (PNG 339 KB)

Supplementary file2 (TIF 679 KB)
