# Supplementary figures and images for: Risk of Recurrence and Cancer Stem Cell Marker CD133 Expression Vary in Males Versus Females with Papillary Thyroid Cancer
Source: Ann Surg Oncol. 2025 Apr 11;32(7):4772–9. doi: 10.1245/s10434-025-17256-2 (PMC12130084; doi:10.1245/s10434-025-17256-2)

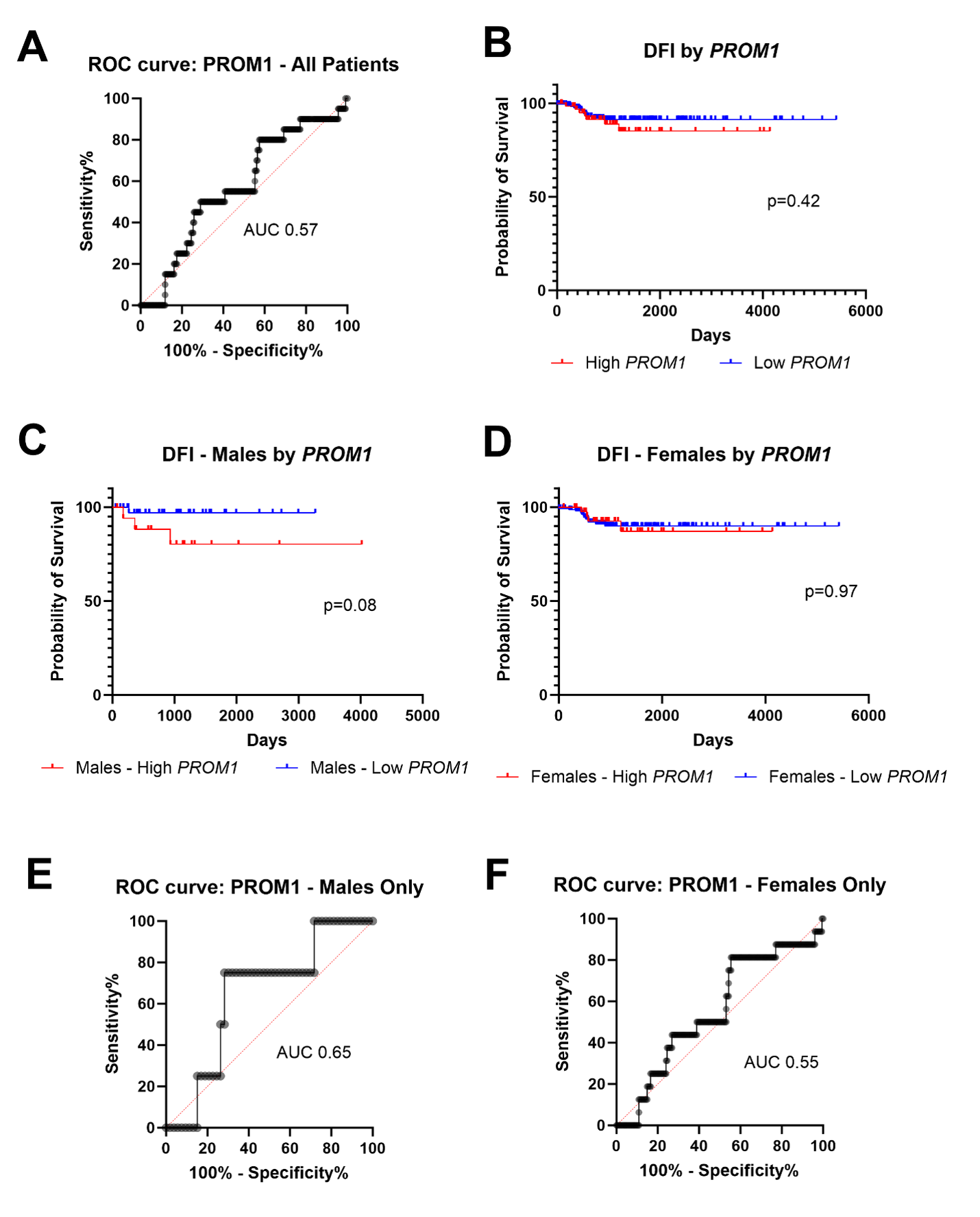

Supplement: Supplementary file 1 — Supplementary file1 (PNG 339 KB) [file 10434_2025_17256_MOESM1_ESM.png]

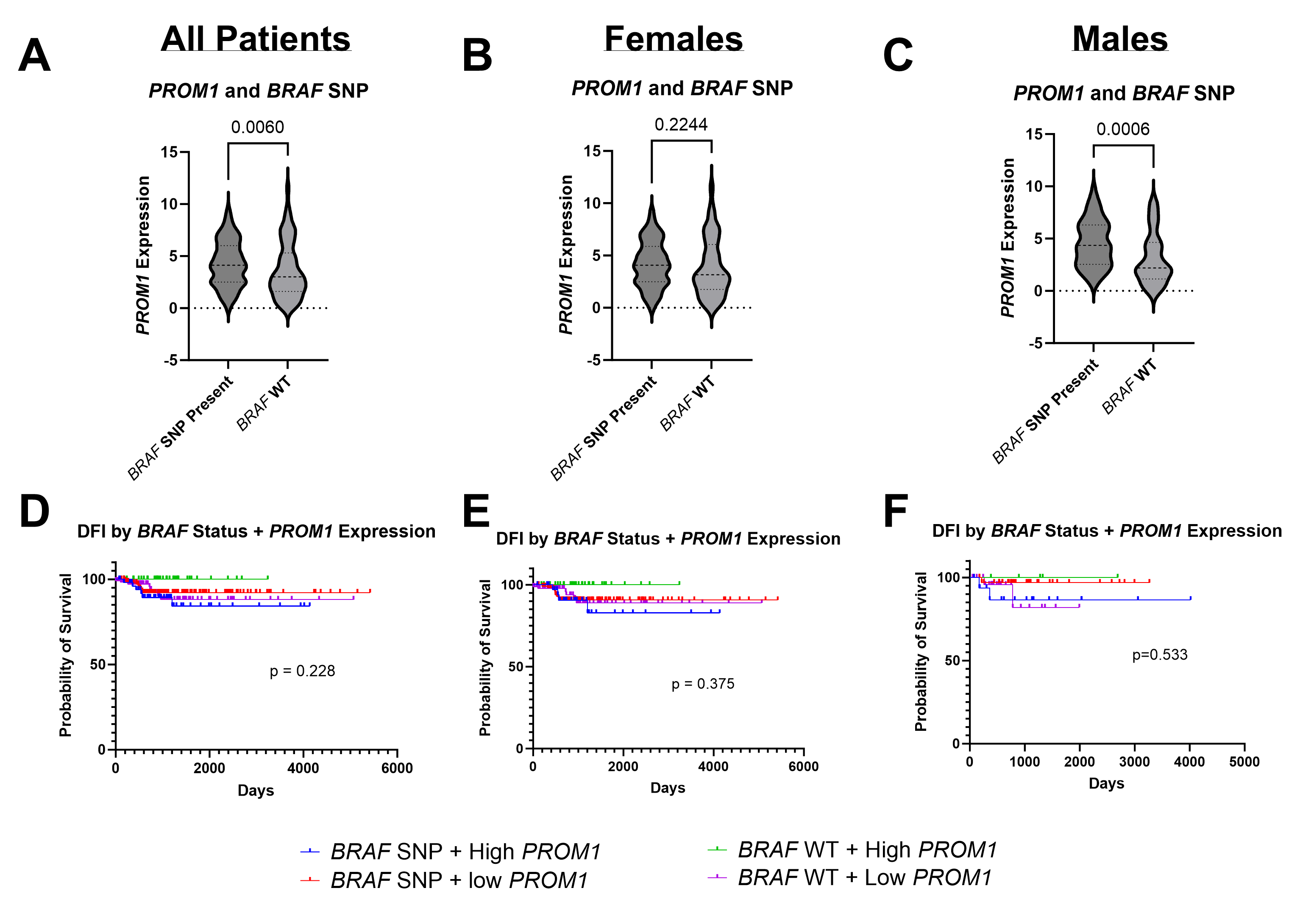

Supplement: Supplementary file 2 — Supplementary file2 (TIF 679 KB) [file 10434_2025_17256_MOESM2_ESM.tif]
